# Nasal Infection of Enterovirus D68 Leading to Lower Respiratory Tract Pathogenesis in Ferrets (*Mustela putorius furo*)

**DOI:** 10.3390/v9050104

**Published:** 2017-05-10

**Authors:** Hui-Wen Zheng, Ming Sun, Lei Guo, Jing-Jing Wang, Jie Song, Jia-Qi Li, Hong-Zhe Li, Ruo-Tong Ning, Ze-Ning Yang, Hai-Tao Fan, Zhan-Long He, Long-Ding Liu

**Affiliations:** Institute of Medical Biology, Chinese Academy of Medical Sciences & Peking Union Medical College, Kunming 650118, China; zhenghuiwen12@126.com (H.-W.Z.); sunming@imbcams.com.cn (M.S.); gl2011@imbcams.com.cn (L.G.); wangjingjing@imbcams.com.cn (J.-J.W.); songjie@imbcams.com.cn (J.S.); lijiaqipumc@yahoo.com (J.-Q.L.); lhz@imbcams.com.cn (H.-Z.L.); ruotongning@126.com(R.-T.N.); zenningyang@163.com (Z.-N.Y.); a779091958@163.com (H.-T.F.)

**Keywords:** enterovirus D68, animal models, ferret, lower respiratory tract pathogenesis

## Abstract

Data from EV-D68-infected patients demonstrate that pathological changes in the lower respiratory tract are principally characterized by severe respiratory illness in children and acute flaccid myelitis. However, lack of a suitable animal model for EV-D68 infection has limited the study on the pathogenesis of this critical pathogen, and the development of a vaccine. Ferrets have been widely used to evaluate respiratory virus infections. In the current study, we used EV-D68-infected ferrets as a potential animal to identify impersonal indices, involving clinical features and histopathological changes in the upper and lower respiratory tract (URT and LRT). The research results demonstrate that the EV-D68 virus leads to minimal clinical symptoms in ferrets. According to the viral load detection in the feces, nasal, and respiratory tracts, the infection and shedding of EV-D68 in the ferret model was confirmed, and these results were supported by the EV-D68 VP1 immunofluorescence confocal imaging with α2,6-linked sialic acid (SA) in lung tissues. Furthermore, we detected the inflammatory cytokine/chemokine expression level, which implied high expression levels of interleukin (IL)-1a, IL-8, IL-5, IL-12, IL-13, and IL-17a in the lungs. These data indicate that systemic observation of responses following infection with EV-D68 in ferrets could be used as a model for EV-D68 infection and pathogenesis.

## 1. Introduction

Recently, there have been many epidemiological studies reporting on the continuous circulation and infection of enterovirus D68 (EV-D68) [1,2]. This virus is associated with severe diseases, including acute respiratory distress syndrome (ARDS) and central nervous system (CNS) clinical signs [3,4,5], and identifying the virulence factors contributing to these manifestations has been the focus of many recent studies [6,7]. EV-D68 was first identified in California in 1962. However, EV-D remains a poorly characterized species of the family *Picornaviridae* [8]. Notably, the mechanisms leading to increased pathogenesis of EV-D68, particularly the tropism infection in the human upper and lower respiratory tract, remain to be discovered. Clinical data has shown that pathology in the patients’ lower respiratory tract (LRT) are mainly featured by serious respiratory diseases in children and acute flaccid myelitis [9,10]. Some studies have shown that the most important pathway of EV-D68 infection is the respiratory tract by binding sialic acid on the membrane [11,12], but the shedding of the virus from the upper respiratory tracts and the early immune response of patient cytokine secretions and excretions have not been well documented. In translational medicine research, the cotton rat models for studying the infection and transfer of EV-D68 can be helpful for characterizing the behavior of the virus as well as physiological responses to it [13]. However, the use of cotton rat-adapted EV-D68 strains for direct intranasal or intraperitoneal inoculation cannot mimic the natural route of infection in humans.

It is well documented that the sialic acid on the surface of respiratory tract can mediate influenza viral receptor binding protein attachment, which is believed to be an important determinant in tissue tropism of this virus [14].For instance, hemagglutinin (HA) of human influenza viruses have a binding preference for α2,6-linked sialic acids (SAs) dominated in upper respiratory tract (URT), supposedly indicating the pathology characteristics in the upper respiratory tract [15]. As an animal model, the domestic ferret (*Mustela putorius furo*) is a conventional model for studying the pathogenesis of viral respiratory infection, including influenza and Severe Acute Respiratory Syndrome (SARS) coronavirus, because there is no need for virus adaptation [16]. Advantageously, ferrets exhibit a similar distribution of sialic acid in respiratory tract as in humans [8,14,17,18]. Due to the respiratory-infecting characteristics of EV-D68 in humans, the infection efficiency of the virus in ferrets could be a key animal model for conducting studies of the EV-D68 infection mechanisms and immune responses via the respiratory tract. In this study, we focused on determining whether or not ferrets have the potential to serve as a small animal model for EV-D68. A nose aerosol spray was used to induce EV-D68 infection in a ferret model, and serial nasal, throat, and feces samples were collected from the ferrets during and after EV-D68 infection. The pathological changes and expressive levels of inflammatory cytokines/chemokines in URT and LRT of ferrets correlated with viral shedding. These results could be useful supporting data for the evaluation of EV-D68-induced manifestations. Collectively, these data provide the rationale to assess the utility of the ferret as a model for EV-D68 infection.

## 2. Materials and Methods 

### 2.1. Ethics Statement and Animal Experiments

The animal experiments in this study comply with the Replacement, Refinement, & Reduction (3R) principles. The study was also in accordance with the Animal Research: Reporting of In Vivo Experiments (ARRIVE) guidelines. The animal procedures were approved by the Institutional Animal Care and Use Committee (IACUC) of the Institute of Medical Biology, Chinese Academy of Medical Sciences. Ferret experiments were conducted, and a total of 18 male ferrets (weight: 800–1000 g) were used in this study. Serum samples from the ferrets were tested by neutralization assay to ensure seronegativity for EV-D68 before the experiments. Fifteen ferrets were infected with 10^4.5^ 50% cell culture infectious doses (CCID_50_) of EV-D68 via the nostrils dropwise, three ferrets of which were included for immune evaluation. Three ferrets used as mock controls were inoculated with 0.5 mL phosphate-buffered saline (PBS). All ferrets were housed in a high-efficiency particulate air-filtered individual isolation unit in an Animal Biosafety Level 2-enhanced (ABSL-2+) facility, which complied with the requirements for ferret housing, environment, and comfort as described in the Guide for Laboratory Animals Care issued by the Institute of Medical Biology.

The animals were visually inspected daily. Body weight and temperature were measured daily for two weeks post-infection. The onset and duration of all visible changes, such as abnormal respiration and excretions, were recorded, as well as any observed sneezing and nasal discharge. Blood samples were collected before and after viral infection. Animal feces, nasal washes, and throat swabs were collected on days 1, 3, 5, 7, 9, 11, 13, and 15 after the challenge and frozen (−80 °C) until analysis. After infection, on days 3, 5, 7, 9, the ferrets were euthanized with an intramuscular injection of a ketamine (20 mg/kg), then the organs or tissues were harvested for histopathology and viral distribution analysis. After the last sacrifice, three ferrets were followed for 28 days to determine the neutralization antibody titer.

### 2.2. Cells and Virus

The EV-D68 Fermon strain used in this study was preserved in the Institute of Medical Biology. EV-D68 seed stocks with a titer of 10^6.5^CCID_50_ units/mL were propagated in Vero cells (ATCC). The seed stocks were diluted to the appointed titer and used for EV-D68 CCID_50_ as well as neutralization antibody assays.

### 2.3. Real-Time PCR Test for Viral Load Quantity

Total RNA was extracted from fresh tissue, feces, bronchoalveolar lavage fluid (BALF), and blood from the experimental animals using the TRNzol-A+ Reagent mini kit (TianGen Biotech, Co., Ltd., Beijing, China) according to the manufacturer’s instructions. The total RNA was eluted in a final volume of 20 µL. For quantification, a single-tube, real-time Taqman RT-PCR assay was performed using the Taqman one-step RT-PCR Master Mix in the CFX96 Touch™ Real-Time PCR Detection system (Bio-Rad, Laboratories, Hercules, CA, USA). The experiments were carried out by adding the primer (200 nm), FAM/TAMRA probe (100 nm) (TAKARA Biotechnology Co., Ltd., Dalian, China), and 2 μL of RNA into the Taqman PCR mater mix, of which total reaction volume is 20μL. The following sequences including EV-D68-specific primer and probe: forward primer, 5′-CACCATACTCACAACTGTGGCAG-3′; reverse primer 5′-CTAGCATTACTGCCTGATTGCCAATG-3′ and the probe 5′-TGACTTGACACTCCAAGCAATGTTTG-3′; The following reaction conditions were applied for all PCR experiments: 5 min at 42 °C and 10 s at 95 °C, followed by 40 cycles at 95 °C for 5 s, and 60 °C for 30 s. A standard reference curve was established by measuring the serially-diluted concentrations of the EV-D68 RNA standards generated from the in vitro transcription of a DNA gene fragment containing the EV-D68 p1 gene region.

### 2.4. Histopathological and Immunohistochemical (IHC) Staining

Tissue samples were obtained from the infected ferrets and were fixed with 10% formalin in PBS and embedded in paraffin. Paraffin-embedded sections were stained with hematoxylin and eosin (H&E). For immunohistochemical detection of the VP1 antigen of EV-D68, slides of paraffin-embedded sections were detected by anti-EV-D68 monoclonal antibodies (GeneTex, Inc., Irvine, CA, USA) and horseradish peroxidase (HRP)-conjugated anti-rabbit IgG antibodies (Cell Signaling Techonology, Shanghai, CST-US subsidiary in China). Peroxidase activity was detected with an Enhanced HRP-DAB Chromogenic Substrate Kit (TianGen Biotech, Co., Ltd., Beijing, China) [19,20].

### 2.5. Laser Confocal Microscopy Analysis of the Infected Ferret Lung Sections

The lung sections were blocked for two hours in 10% normal goat serum to reduce nonspecific antibody binding. The lung was then incubated with anti-EV-D68VP1 antibody 5 μg/mL (GeneTex, Inc., Irvine, CA, USA) and with biotinylated MAA (α2,3-linkage) and SNA (α2,6-linkage) 5 μg/mL (Vector Laboratories, Inc., CA, USA) at 4 °C overnight. The sections were rinsed extensively in Tris-buffered saline. The cell nuclei were strained by DAPI (Beyotime Biotech, Co., Ltd., Shanghai, China). The primary antibody was detected using 5 μg/mL of Fluorescein Avidin DCS (Vector Laboratories, Inc., Burlingame, CA, USA) and 2 μg/mL Texas-Red-conjugated goat anti-rabbit IgG antibodies (Molecular Probes, Carlsbad, CA, USA) [20,21]. The stained slides were analyzed under a Leica TCS SP8Laser Confocal microscope (Leica Microsystems, Wetzlar, Germany).

### 2.6. Cytokine Quantification by Magnetic Beads-Based Bio-Plex Assay

In this study, the Non-Human Primate Cytokine Magnetic Bead Panel Kit (Millipore Corporation, Billerica, MA, USA) is attempted for simultaneous quantification of the infected ferret serum of the following 23 kinds of cytokines: G-CSF, GM-CSF, IFN-γ, IL-1B, IL-1a, IL-2, IL-4, IL-5, IL-6, IL-8, IL-10, IL-12/23, IL-13, IL-15, IL-17A, IL-18, MCP-1, MIP-1B, MIP-1a, sCD40L, TGF-a, TNF-a, and VEGF. In brief, 25μL aliquots of standard, control and sample were diluted 1:4 with diluent, following incubation with antibody-coupled beads, and thoroughly washed, the detection antibodies were added for co-incubation and testing. Finally, the plate was run on a Bio-PLEX (Bio-Rad, Laboratories, Inc.). A five-parameter logistic method was used to calculate analytic concentrations in samples.

### 2.7. Quantification of Cytokine mRNA

RNA isolations were performed on ferret lung tissue samples with the TRNzol-A+ Reagent kit (TianGen Biotech, Co., Ltd., Beijing, China) according to the manufacturer’s protocols. Then, cytokine expression levels were normalized to Beta-actin (β-actin) and are reported as the fold change compared with mock-infected animals. Primer sequences for IL-1a, IL-5, IL-8 [22], IL-12 [23], IL-13, IL-17A, and β-actin [23] were published elsewhere. Primers sequences for the remaining genes are as follows: IL-1a: forward, 5′-GAGATGCCTGAGACACCCAAA-3′; reverse, 5′-TGTGCACCAGTTTTCGTTCC-3′; IL-5: forward, 5′-GGAGGCTGTGGATAAACTATTCC-3′; reverse, 5′-CCGGTGTCCACTCAGTGTTTAT-3′; IL-13: forward, 5′-AGAATCAGGCATCCCTCTGC-3′; reverse, 5′-CTTACTGGAGATCCCTGCCG-3′; IL-17A: forward, 5′-GTGCTGACGGGACGGTAAA-3′; reverse, 5′-ACCAGCATCTTTTCCAACCG-3′; Quantitative real-time PCR (qRT-PCR) was performed by using aCFX96 Touch^TM^ Real-Time PCR Detection system (Bio-Rad, Laboratories), and a One Step SYBR PrimeScript^TM^ RT-PCR Kit (TAKARA Biotechnology Co., Ltd.).Each reaction consisted of 1 cycle of 42 °C for 5 min, 95 °C for 10 s, followed by 40 cycles of 95 °C for 5 s and 60 °C for 30 s.

### 2.8. Neutralization Antibody Titer Test

In brief, ferret serum was heat-inactivated for 30 min at 56 °C, then diluted 1:2 in minimum essential medium containing 2% fetal bovine serum (FBS)(Gibco, LifeTechbologies, Shanghai, China). After cooling, the medium to room temperature, diluted serum was transferred in triplicate to the first row of one 96-well plate and then diluted two-fold from 1:4 to 1:512. One hundred CCID_50_ were combined with the diluted sera in a 96-well, white, opaque-bottom plate and incubated at 35 °C for 3 h before adding 10,000 Vero cells/well. After incubating the samples for six days at 35 °C and 5% CO_2_, the reciprocal measurement of the highest serum dilution that inhibited 50% of the viral cytopathic effect was defined as the neutralization antibody (NA) titer against the relative EV-D68. Neutralization titers were estimated with the Spearman–Karber method and expressed in log2 form (e.g., 4 is a titer of 1:16) [24,25].

### 2.9. Statistics

GraphPad Prism 5 (Version 5.0, La Jolla, CA, USA) was used to graph data and perform statistical analyses. To compare blood cell count and cytokine expressed level between groups, the Mann–Whitney U test was used. Mean ± SEM (standard error of the mean) were graphed and * (*p* < 0.05) was considered to be statistically significant.

## 3. Results

### 3.1. EV-D68 Infection in Ferrets Caused Normal, Cold-Like Clinical Signs

**Figure 1:**
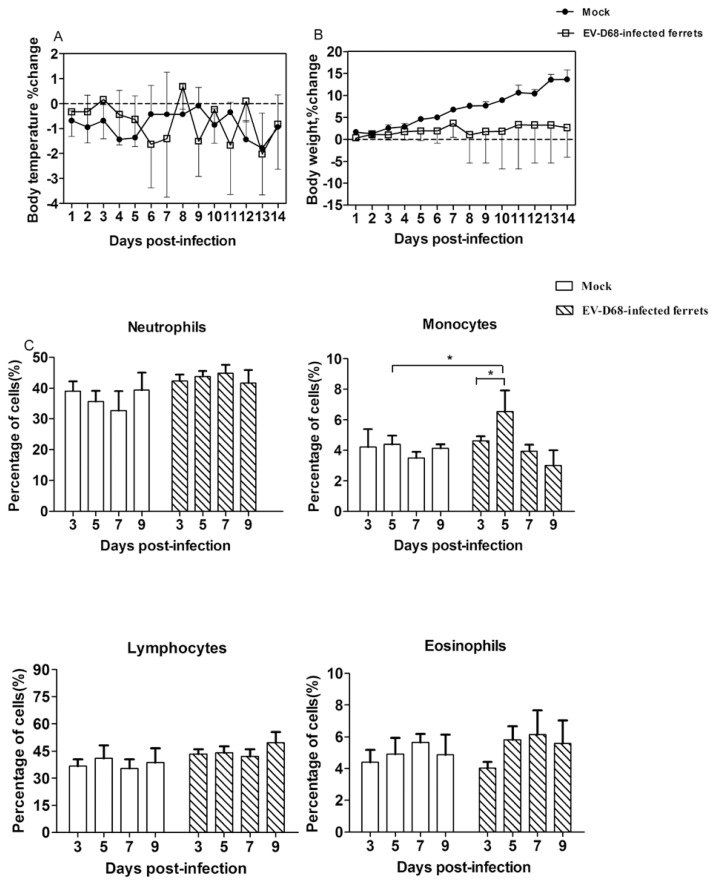
Clinical features of ferrets infected with EV-D68 virus. 12 EV-D68-infected ferrets (10^4.5^CCID_50_ per animal) and 3 mock-infected ferrets (equivalent volumes of virus-free DMEM) were monitored daily for clinical features. The data were recorded as percentage changes compared with value of zero days post-infection. (**A**) The body temperature changes of infected and uninfected animals. Data is mean of rectal temperatures of each ferret groups of 3 to 12 ferrets per time point. The error bars show the SEM of temperature changes at different time; (**B**) The body weight changes of infected and uninfected animals. Data is mean of each groups of 3 to 12 ferrets per time point. The error bars show the SEM of temperature changes at different time; (**C**) The complete blood count (CBC) analysis of mock and EV-D68-infected ferrets. The percentages of lymphocytes, monocytes, neutrophils, and eosinophils were plotted respectively. Blood samples were collected at three, five, seven, and nine days post-infection. All samples were run in triplicate, with mean value and SEM. Monocytes: *, *p* < 0.05, EV-D68-infectedferrets compared with mock on five days post-infection; five days post-infected ferrets compared with three days post-infected ferrets.

Generally, patients infected with this virus can appear to have various disease severities ranging from mild respiratory illnesses, such as cold-like clinical signs, to severe lower respiratory tract infections (LRTI), including pneumonia, wheezing, and bronchiolitis [26]. In our study, the clinical signs of respiratory illness, including cough, nasal discharge (Figure S1A) and dry nose (Figure S1B) were present in 4 of 15 infected animals. No significant increase in body temperature (Figure 1A) was observed in the ferrets with virus infection, while this phenomenon is in accordance with the clinical report that some patients with EV-D68 infection were characterized by low-grade or absent fever [27,28]. Although, there is an increase in all ferret body weight (Figure 1B) during the period of experimental observation, the uninfected ferrets gained more weight than the infected ferrets (a mean of 13.7% vs. 2.7%) at 14 days post-infection, indicating that the overall health of the mock ferrets was better. In addition, during early infection (5–7 days post-infection), some ferrets with EV-D68 infection had a slightly increase of neutrophils (from mean 42.3% to 44.6%) and monocytes (from mean 4.6% to 6.5%) when compared with the three days post-infection ferrets, but there is no change in the number of the lymphocytes and eosinophils (Figure 1C).

### 3.2. Virus Shedding Potential and Distribution in Different Tissues of EV-D68-Infected Ferrets

**Figure 2:**
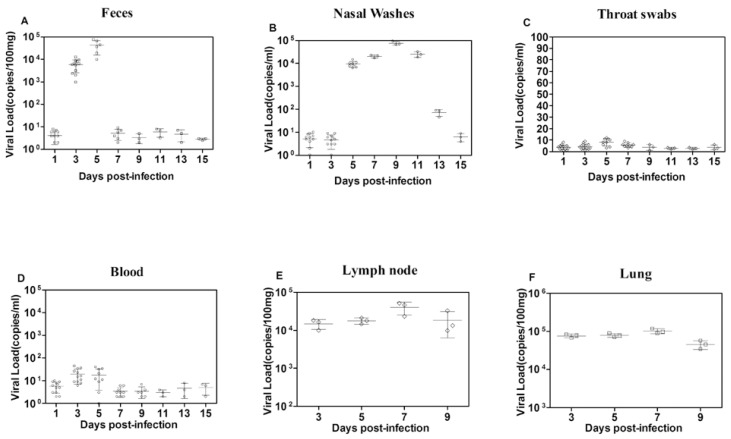
Dynamic distribution of EV-D68 virus in infected ferrets through respiratory route. Twelve ferrets were infected with EV-D68 (10^4.5^CCID_50_/ferret) via nostrils dropwise and three ferrets set as mock control. Viral load in feces (**A**) and nasal washes (**B**) and throat swabs (**C**) of infected ferrets were detected in the whole course of EV-D68 infection (1, 3, 5, 7, 9, 11, 13, 15 days post-infection); (**D**) Viral RNA was picked up from blood samples and analyzed by Taqman real-time quantitative PCR assay, according to the protocols provided by the qPCR kit. Each blood samples were gathered during the whole EV-D68 infection course (1, 3, 5, 7, 9, 11, 13, 15 days post-infection); (**E**)Viral load in lung of ferret at different day post-infection (3, 5, 7, 9 days post-infection); (**F**) Viral load in lymph nodes at different day post-infection(3, 5, 7, 9 days post-infection). The vitro-synthesized RNA was applied to quantify the viral copies of each RNA sample, and viral RNA’s relative copies for each sample was calculated by the mathematical formula as follow: [(μg of RNA/μL)/(molecular weight)] × Avogadro’s number = viral copy number/μL. A viral load which is less than 10 copies is regarded as negative.

Similar to other enteroviruses, EV-D68 has the ability to infect lymphocytes [29]. In addition, several epidemiological studies have demonstrated that EV-D68 is associated with severe lower respiratory tract infection and central nervous system (CNS) pathogenicity, including acute focal limb weakness, paralysis, and acute cranial nerve dysfunction [4,27,30]. In our research, the partial VP1 gene (120 nt) was detected using RT-PCR analysis with sequence-specific primers and probes in the feces, nasal washes, throat swabs, blood, lung, BALF, trachea, and CNS samples that were collected at different times post-infection from the ferrets. The virus was detected in the feces and nasal washes from the third day post-infection, and a peak level of over 70,000 copies per 100 mg sample (Figure 2A), was reached on the fifth day, and 90,000 copies per 1 mL of nasal wash (Figure 2B) was detected on the ninth day, but no virus was detected in the throat swabs (Figure 2C). For viremia of EV-D68 infection, the viral load in blood samples is less than 50 copies/mL (Figure 2D). Several studies have indicated that the enterovirus invasion lead to a transient minor viremia that delivers the virus to lymphoid tissues [31]. In this study, the virus replication in the axillary lymph nodes was detected at five and seven days post-infection. With levels of 18,000–50,000 copies per 100 mg of tissue (Figure 2E). To measure the profile of virus replication in lower respiratory systems, the EV-D68 virus was detected in lung, trachea, and BALF. Infectious viruses were detectable from three to seven days post-infection. With a peak level of 89,000 copies per 100 mg of lung tissue on the fifth day post-infection (Figure 2F). However, viral load in BALF and in the trachea was less than 40 copies/mL (Figure S2A,B). Detection of EV-D68 Fermon strain was negative in CNS (including brain, midbrain, cerebellum, and medulla oblongata) (Figure S2C). Although some clinical results report that there is a possible association between EV-D68 and neurological disease, we did not observe the central nervous system symptoms in ferrets [4], which was in accordance with Schieble’s experimental result from sucking mice by inoculation with four strains of EV-D68, in which CNS impairments were observed only in mice infected with the Rhyne strain—not in Fermon, Franklin, and Robison strains [32].

### 3.3. Histopathological Examinationand Immunohistochemical Analysis

**Figure 3:**
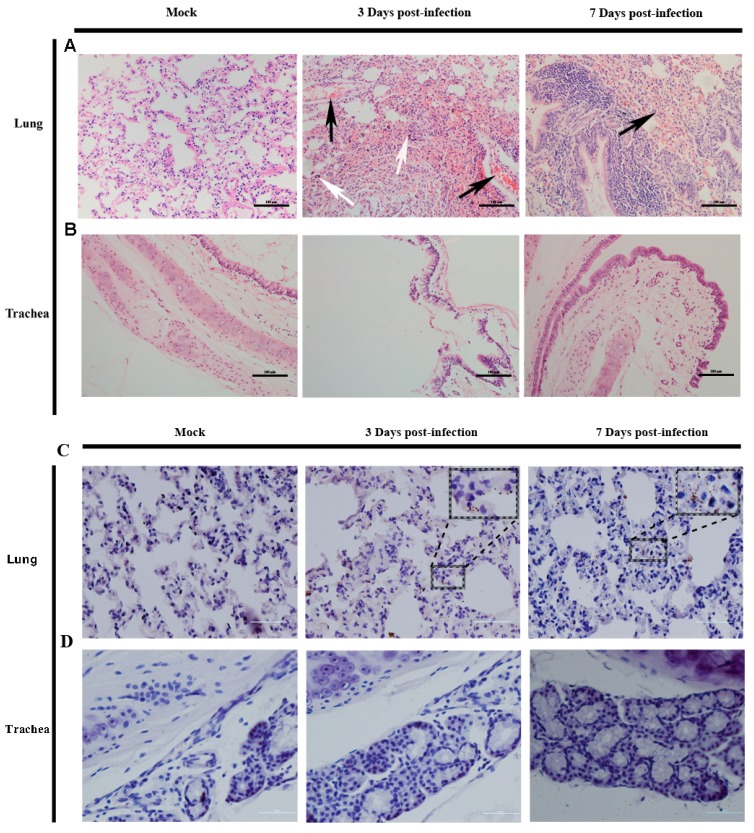
Pathological manifestations in respiratory apparatus of EV-D68-infected ferrets. (**A**) The clinical pathological features of EV-D68 infection in ferret lung tissues; (**B**) There is no pathological change in trachea. In these images, the white arrow represents the inflammatory cell infiltration, the black arrow points the diffuse alveolar hemorrhage. The microscope magnified the images 200 times, Bar, 100 µm; (**C**) The viral antigen expression in lung tissue from ferrets infected by 10^4.5^CCID_50_ EV-D68. The black dashed boxes indicate the EV-D68 VP1 antigen expression and the insert picture enlarge the antigen (on the top right corner); (**D**) There is no VP1 antigen detectable in trachea. Images are magnified by 400 times; Bar, 50 μm.

Two clinical cases have reported that diffusion and patchy alveolar infiltration were present in lung tissues of the EV-D68-infected patients [10,33] according to computed tomography angiograms of the chest. In this study, the tissue samples from the trachea and lungs were histopathologically examined in order to observe the pathological change of the respiratory apparatus from the ferret infected with the EV-D68. Inflammation and diffuse alveolar hemorrhage were found in lungs where the viral load level was very high from EV-D68-inoculated ferrets on three and seven days post-infection (Figure 3A), but there were no pathological changes in the trachea (Figure 3B). These findings suggest that ferret infection might have a remarkable pathogenesis in the lower respiratory tract at three to seven days post-infection (Figure 3A). At the same time, immunolabeling of the VP1 antigen was observed in lung cells around the pulmonary alveolus cells (Figure3C), while VP1 antigen is undetectable in the trachea (Figure 3D). Together with the virus detection and histopathological analyses of the lung tissues, all EV-D68-infectedanimals likely demonstrated pulmonary manifestations at three to seven days post-infection.

### 3.4. Confocal Imaging of EV-D68 VP1 with the α2,6-Linked SAs in the Lung Tissue of EV-D68-Infected Ferrets

**Figure 4:**
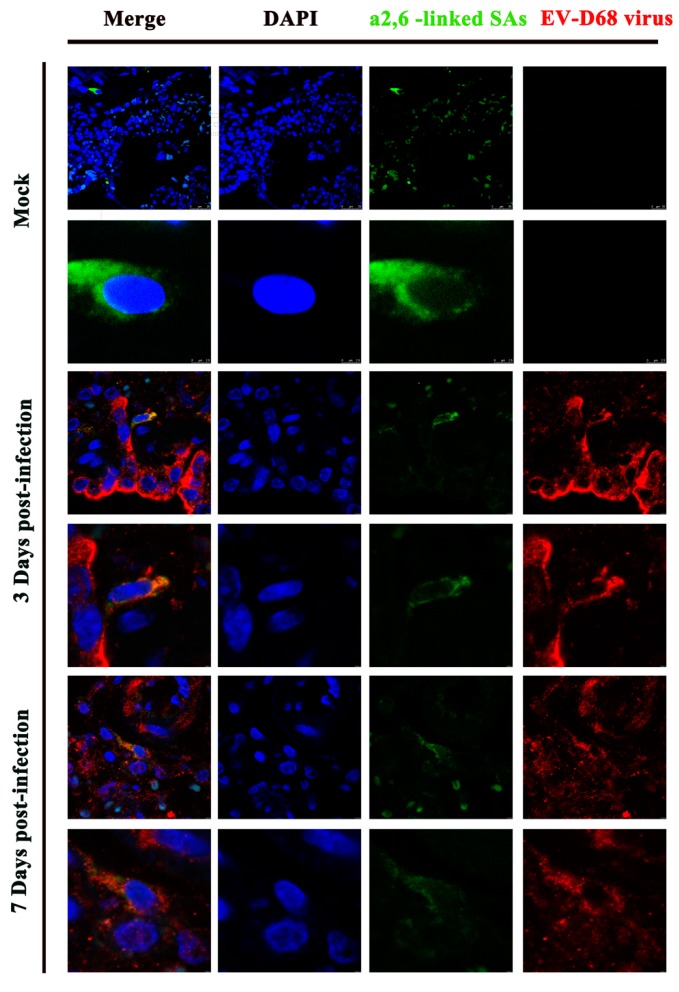
Confocal imaging of α2,6-linked SAs and viral antigen in the lung tissue of EV-D68-infected ferrets. The samples were obtained at three and seven days post-infection. EV-D68 VP1 antigen was labeled with Texas-Red-conjugated anti-IgG antibody (red), while α2,6-linked SAs were labeled with FITC (green). Images are shown at 630× magnification.

Sialic acid (SA) has been reported as the receptor for EV-D68, and it has been shown that EV-D68 has a stronger affinity for α2,6-linked SAs than for α2,3-linked SAs [12,34]. To compare the distribution of the α2,3-linked SAs and α2,6-linked SAs in the cells of lungs of viral-infected animals, the lung samples were stained by anti-EV-D68 virus VP1 protein monoclonal antibodies, then were detected by a Texas-Red-conjugated secondary antibody (Figure 4). The biotinylated lectins SNA and MAA were used to detect the α2,6-linked SAs and α2,3-linked SAs, respectively. The bound lectins were distinguished by avidin DCS labeled by FITC (Figure 4 and Figure S3). In the ferrets, we found that α2,3-linked SAs are more dominantly expressed in the lung tissue (Figure S3) than α2,6-linked SAs (Figure 4), However, both three days post-infection and seven days post-infection, the overlaid image of the two colors (green and red) exhibit intense co-location of the α2,6-linked SAs on EV-D68-positive lung cells (Figure 4). Remarkably, α2,3-linked SAs were not observed on the EV-D68 positive lung cells according to the co-localized image (Figure S3).These results indicated EV-D68 infection may prefer α2,6-linked SAs over α2,3-linked SAs in the ferrets’ lower respiratory tracts (Figure 4).

### 3.5. Inflammatory Cytokines Increased in Pulmonary Pathogenesis

**Figure 5:**
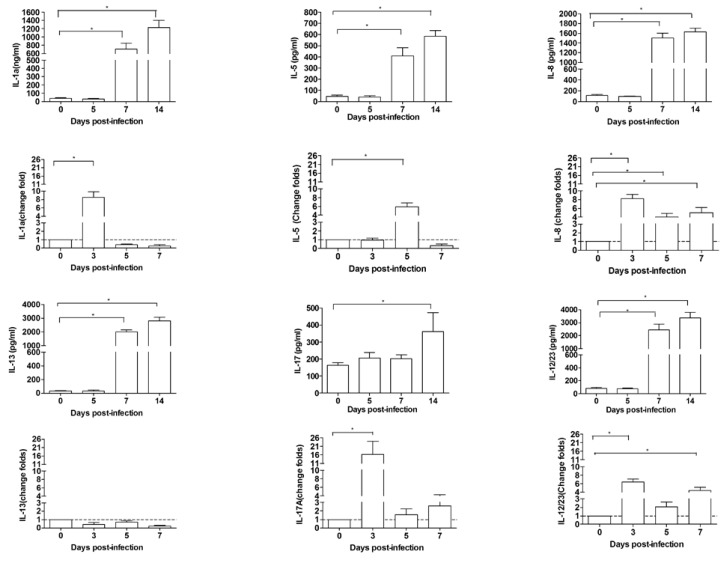
Inflammatory cytokines detection of the ferrets after EV-D68 infection. (**A**) Cytokines (IL-1a, IL-5, IL-8) of serum were determined using multiplex bead-based Bio-Plex assay and are detected at different day (0, 5, 7, 14) post-infection; (**B**) The average transcription levels of interleukin-1α (IL-1α), IL-5, IL-8 of lungs were determined by qPCR and are plotted graphically for various time points (0, 5, 7, 9) following infection. Increases of mRNA levels were relative to β-actin and then normalized to the PBS control groups; (**C**) Cytokines (IL-12, IL-13, IL-17a) of serum were determined using multiplex bead-based Bio-Plex assay and are detected at different days (0, 5, 7, 14) post-infection; (**D**) The average transcription levels of IL-12, IL-13, IL-17a of lungs were determined by qPCR and are plotted graphically for various time points (0, 5, 7, 9) following infection. Increases of mRNA levels were relative to β-actin and then normalized to the PBS control groups. Average data were obtained from three independent experiments, and the error bars indicate SEM. The dot line represents the mRNA base level. Horizontal bars show the statistical analysis performed between the selected two groups. *, *p* < 0.05. The expressed level of different cytokine on 7 & 14 days post-infection compared with 0 days post-infection.

To determine the possibility of the pathological progress in the LRT of EV-D68-infected ferrets due to acute lung injury of inflammatory immune responses, we intended to evaluate the inflammatory cytokine levels in the lung during the early and late stages of infection. Despite the prominent use of ferrets in medical research, the immune system of ferrets remains poorly characterized. There are insufficient ELISA reagents for the detection of cytokines in ferrets, so we attempted to use the Bio-Plex Suspension Array System to evaluate serum cytokine levels. Numerous studies have reported that many animal species’ antibodies specific for cytokines can be used to detect ferret cytokines [35,36]. Therefore, in this study, the non-human primate cytokine Th1/Th2 assay was used to cross-test 23 cytokines and chemokines in ferrets following the protocol in the user manual (Figure S4). For verification of this test, the levels of gene expression were also measured by qRT-PCR. The results indicated differential expression of the inflammatory cytokines/chemokines in the LRT, showing high expression levels of interleukin-1α (IL-1α), IL-5, IL-8 (Figure 5A,B), IL-12, IL-13, and IL-17a in serum (Figure 5C,D), which correlated with the peak levels of viral shedding and pathological changes in the lungs. These data suggest that infection increased the release of inflammatory cytokines in the infected ferret lungs during early infection and lung pathology was continuously exacerbated during the progression of infection and caused lung edema as well as lung injury at the middle and late stages of infection.

### 3.6. Immunological Response of Ferrets after EV-D68 Infection

**Figure 6:**
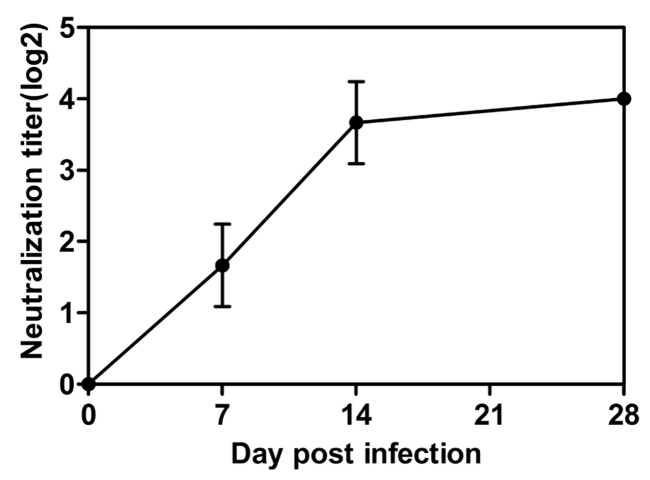
The neutralization antibody titer of ferrets against the EV-D68 virus. Each plot represents the average of three logarithmically transformed measurements (log2).Vertical bars represent the mean value ± SEM. The number under each figure represents the timing of blood collection at different days after the inoculation.

To detect the ability of viral infection to elicit an antibody response, three ferrets were followed for up to 28 days after inoculation. The immunological analysis of the EV-D68-infected animals showed a typical antibody response of viral-induced characteristics. The neutralization antibody GMT exhibited a slight and slow increase of 1:16 levels at week 4 after inoculation (Figure 6). With respect to the report by Patel et al., in the EV-D68 Cotton rat model, Fermon infection resulted in no detectable neutralization antibody (NA) response [13]. Findings in this study demonstrate that the EV-D68 Fermon strain infection may induce faint immunological response in the form of the neutralization antibody titer in ferrets. 

## 4. Discussion

To determine the in vivo effects of viral replication and pathogenesis, mice and rat cotton models have been used to study EV-D68 infection [13,32]. In these studies, cotton rats infected nasally with 10^6^CCID_50_ of the EV-D68 Fermon strain demonstrated a relatively weak infection and replication profile with non-obvious signs of pathological changes [13]. However, the ability to systematically evaluate infection progress in small rodents is limited, and such models do not yield sufficient pathogenic and pathological evidence for infection analysis. Therefore, the implementation of effective ferret models now represents an obligatory step for the preclinical evaluation of respiratory pathogen infections [37,38]. With the similarity of the distributing profile in the human respiratory tract α2,6-linked SAs of ferrets were dominant in the epithelia of the URT, and a relative increase of α2,3-linked SAs in LRT, along with the continued presence of α2,6-linked SAs [14], ferrets have largely been used to study respiratory infections, such as influenza, respiratory syncytial virus, and SARS virus. The infection pathways of EV-D68 were reported to be in the respiratory tract [39,40], indicating that it would be easier to induce respiratory infections in ferret models, which would display symptoms similar to those in humans.

In this study, we used a ferret model to assess clinical symptoms, viral replication, and shedding, pathogenicity, and expression of inflammatory cytokines in the respiratory tracts of ferrets infected with the EV-D68 virus. This model utilized a commonly-employed intranasal challenge route for ferrets to assess the natural mucosal routes of infection. In the results, we found that there was a clear profile in viral replication rates in ferrets compared to results with cotton rats described by Patel [13]. Notably, despite having direct access to the nose upon infection, we observed less viral shedding from nasal discharges compared to fecal matter. Instead, the most impressive viral burdens were noted in the lung, which were similar to the experimental results for cotton rats [13]. While the EV-D68 infection was not measured directly by viral titration in our study, the results of qRT-PCR for viral genome and immunohistochemical analysis for viral antigens can support the EV-D68 virus replication in infected ferrets. Moreover, since EV-D68 usually targets the respiratory tract for infection and induces severe pneumonia among children who have asthma or a history of wheezing [41,42], we also focused on analyzing the pathogenesis in the URT and LRT tissues. Our results clearly showed inflammation and diffuse alveolar hemorrhage occurred in the lungs of EV-D68-inoculated ferrets at three and seven days post-infection, suggesting that the ferret infections might have remarkable pathogenesis in the lower respiratory tract.

Despite of initializing the common cold-like symptoms (e.g., a runny nose, sore throat, and cough) in the upper respiratory tract, more serious pneumonia-like symptoms related with the lower respiratory tract may occur in some cases [26,43]. Thus, EV-D68 attaching and invading in the LRT could contribute to above outcomes. Furthermore, we investigated the relationship between lectin receptors on the surface of lower respiratory tract cells and EV-D68 invasion. In our findings, α2,6-linked SAs staining was more apparent with viral infection, but less of a signal appeared in the α2,3-linked SAs, which indicated that EV-D68 specifically recognized the α2,6-linked SAs of cell tropism in the ferret lower respiratory tract, inducing LRT EV-D68 infection and pathogenic changes. In this case, ferrets are proposed to be a good small-animal model of EV-D68-related lower respiratory manifestations.

Inflammatory immune responses have been reported to be related to acute lung injury after viral infection. We further assessed the inflammatory cytokine and chemokine levels during pathological changes in the LRT of EV-D68-infectedferrets. In the serum and lung during the early stages of infection, there was increased expression of inflammatory cytokines/chemokines, including interleukin-1α (IL-1α), IL-5, IL-8, IL-12, IL-13, and IL-17a, which correlated with the pathological changes in the lungs and may play a key role in causing lung edema as well as lung injury in the middle and late stages of infection.

Taken together, these data imply that the ferret models have the potential to be used for characterizing key events in the pathogenesis of EV-D68, even for the Fermon strain, which has lower antigenicity. This model may be useful for evaluating the newly-isolated EV-D68 strains and potential candidate medical interventions, including vaccines. Further studies are needed to more fully characterize the transmission of infection in ferrets to expand the predictive efficacy of this model for intensive study.

## Supplementary Materials

The following are available online at www.mdpi.com/1999-4915/9/5/104/s1. Figure S1: Clinical symptoms of the ferrets infected by EVD68 via the respiratory route, Figure S2: Virological analysis of BALF, trachea, and other tissues of ferrets, Figure S3: Confocal imaging of α2,3-linked SAs and viral antigen in the lung tissue of EV-D68-infectedferrets, Figure S4: Twenty-three kinds of cytokines detected in the ferrets after EV-D68 infection.
