# Supplementary material for: Nasal Infection of Enterovirus D68 Leading to Lower Respiratory Tract Pathogenesis in Ferrets (Mustela putorius furo)
Source: Viruses. 2017 May 10;9(5):104. doi: 10.3390/v9050104 (PMC5454417; doi:10.3390/v9050104)
Supplement: Supplementary file 1 [file viruses-09-00104-s001.pdf]

Supplementary Materials: Nasal Infection of Enterovirus D68 Leading to Lower Respiratory Tract Pathogenesis in Ferrets (*Mustela putorius furo*)

Hui-Wen Zheng, Ming Sun, Lei Guo, Jing-Jing Wang, Jie Song, Jia-Qi Li, Hong-Zhe Li, Ruo-Tong Ning, Ze-Ning Yang, Hai-Tao Fan, Zhan-Long He and Long-Ding Liu

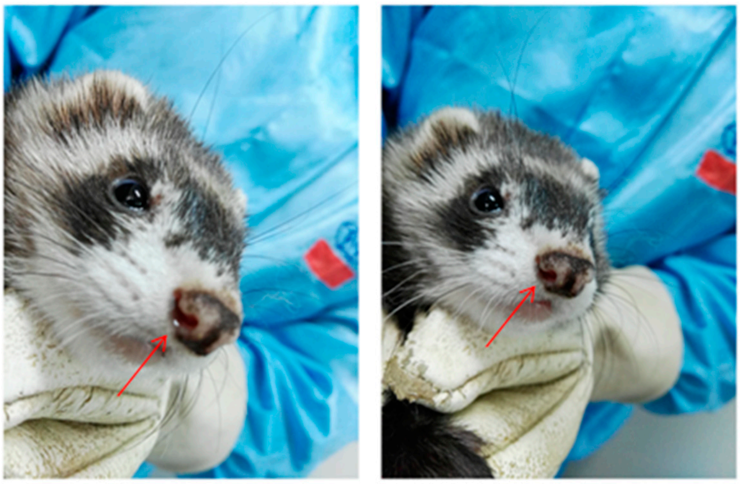

(A) (B)

Figure S1. Clinical symptoms of the ferrets infected by EV-D68 via respiratory route. (**A**) postnasal drips; (**B**) drynose tips were observed in the infected ferrets (red arrow).

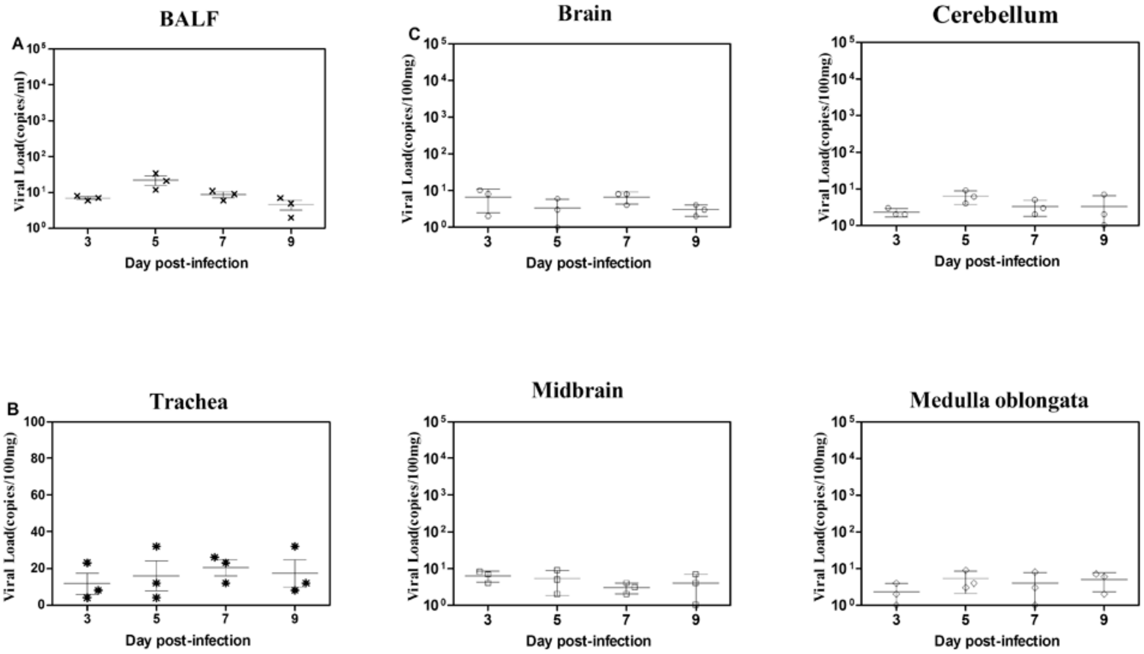

Figure S2. Virological analysis of BALF and trachea and other tissues of ferrets. Ferrets were sacrificed after infection on 3, 5, 7, 9 day. Viral loads were depicted in (**A**) BALF and (**B**) Central Nervous System (CNS) (including brain, midbrain, cerebellum, and medulla oblongata). The viral load which is less than 10 copies is regarded as negative.

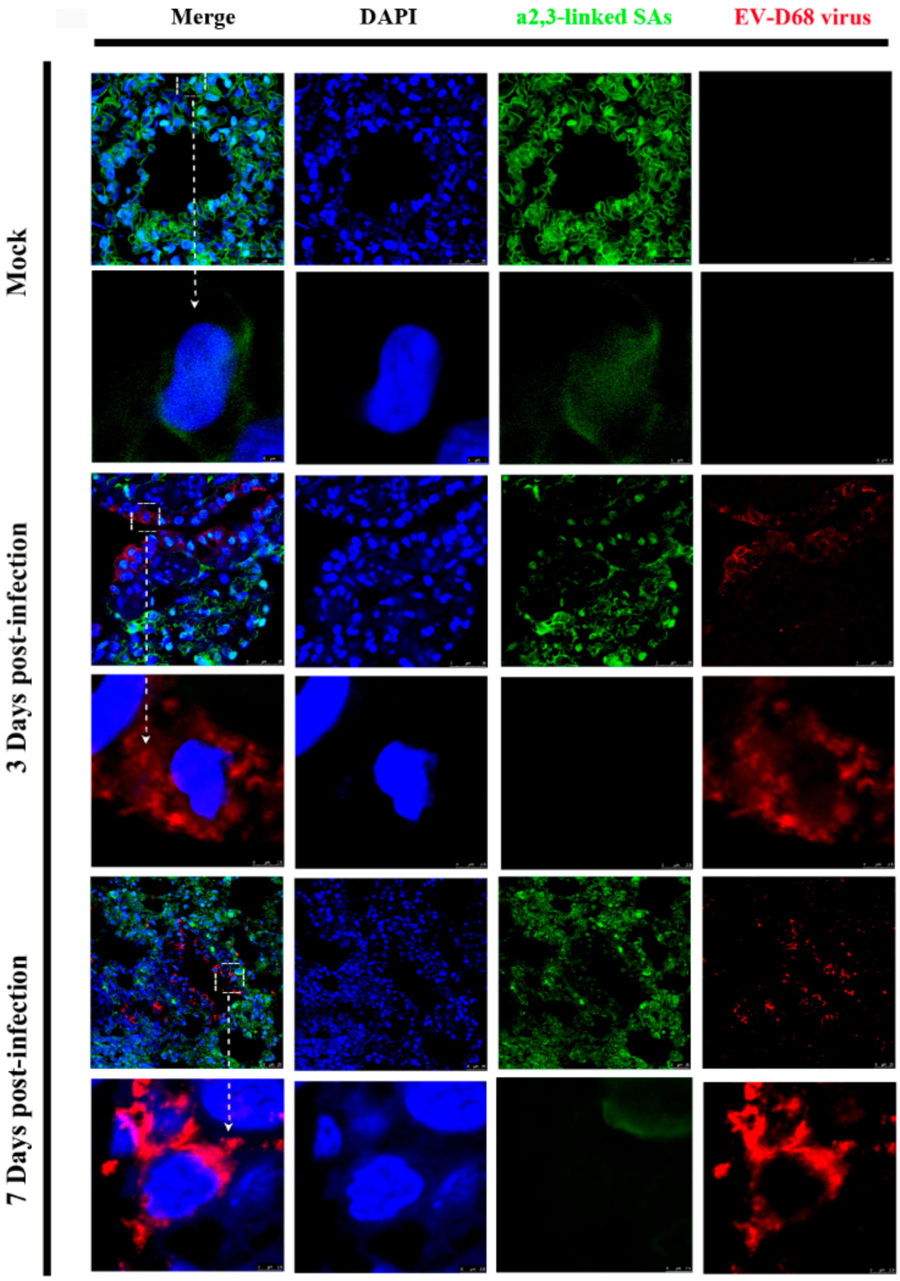

Figure S3. The ferrets were infected with the EV-D68 virus. At 3 and 7 days after infection. The lung tissues were labeled with anti-EV-D68 VP1mAb and visualized with Donkey Anti-Rabbit IgG Texas-Red-conjugated secondary antibodies (Red), while α2,3-linked SAs were labeled with FITC (green). Images are shown at 630 × magnification.

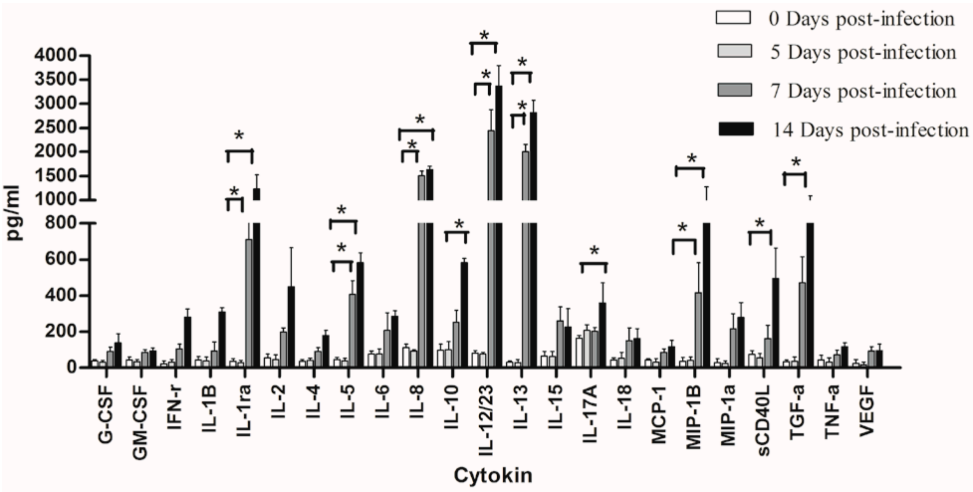

FigureS4. Bio-Plex assay performance in Serum. Serum concentrations of 23 kinds of cytokine (G-CSF, GM-CSF, IFN-γ, IL-1B, IL-1a, IL-2, IL-4, IL-5, IL-6, IL-8, IL-10, IL-12/23, IL-13, IL-15, IL-17A, IL-18, MCP-1, MIP-1B, MIP-1a, sCD40L, TGF-a, TNF-a, VEGF). Comparison of the cytokine response at different days post-infection (0, 5, 7, 14 days post-infection). Each value represents the mean of at least three ferrets. Error bars indicate the SEM. '* $p < 0.05$. '*, $p < 0.05$ the expressed level of different cytokine on 7 &14 days post-infection compared with 0 days post-infection.

© 2017 by the authors; licensee MDPI, Basel, Switzerland. This article is an open access article distributed under the terms and conditions of the Creative Commons by Attribution (CC-BY) license (http://creativecommons.org/licenses/by/4.0/).
